# What Is COVID-19 Teaching Us About Community Health Systems? A Reflection From a Rapid Community-Led Mutual Aid Response in Cape Town, South Africa

**DOI:** 10.34172/ijhpm.2020.167

**Published:** 2020-09-01

**Authors:** Manya van Ryneveld, Eleanor Whyle, Leanne Brady

**Affiliations:** ^1^School of Public Health, University of the Western Cape, Cape Town, South Africa.; ^2^Health Policy and Systems Division, School of Public Health and Family Medicine, University of Cape Town, Cape Town, South Africa.

**Keywords:** Community Health Systems, Collective Action, Mutual Aid, COVID-19, South Africa

## Abstract

The coronavirus disease 2019 (COVID-19) pandemic has exposed the wide gaps in South Africa’s formal social safety net, with the country’s high levels of inequality, unemployment and poor public infrastructure combining to produce devastating consequences for a vast majority in the country living through lockdown. In Cape Town, a movement of self-organising, neighbourhood-level community action networks (CANs) has contributed significantly to the community-based response to COVID-19 and the ensuing epidemiological and social challenges it has wrought. This article describes and explains the organising principles that inform this community response, with the view to reflect on the possibilities and limits of such movements as they interface with the state and its top-down ways of working, often producing contradictions and complexities. This presents an opportunity for recognising and understanding the power of informal networks and collective action in community health systems in times of unprecedented crisis, and brings into focus the importance of finding ways to engage with the state and its formal health system response that do not jeopardise this potential.

## Re-imagining the Community Health System

 Coronavirus disease 2019 (COVID-19) has had major implications for how people around the world are thinking about, experiencing and re-imagining their health systems, in particular at the community level. The nature of the disease, as well as the implementation of hard lockdowns, travel bans and restrictions on local movement, has meant that its impact has been felt by virtually every person in the world. In South Africa, as elsewhere, COVID-19 has provoked swift and wide-ranging mobilisations of both state and civil society. Amongst the latter are legal and rights-based initiatives, pressure groups for increased social security allocations, non-governmental organisations providing various forms of relief and a number of mutual aid initiatives that have sprung up across many of the nine provinces. In this piece we explore lessons from the emergence of a community action network (CAN) in Cape Town in response to COVID-19, known as Cape Town Together (CTT), in which the authors have been active participants. These insights have emerged out of processes of collective sensemaking enabled through ZOOM conversations, WhatsApp groups discussions, and joint actions or projects that the authors have been a part of. As embedded researchers in this community led response we hope to share some initial insights to spark conversation on the network’s forms of organising and on the contested space of interaction, negotiation and potential for learning that results when this kind of bottom-up approach meets the range of other responses, especially that of the state and its formal health system.

We draw on definitions of the community health system as a complex, overlapping set of systems, involving actors spanning various societal levels and concerned with the production of health at the interface between top-down building blocks of the health system and bottom-up mobilisation of community responses.^1,2^ Community-level responses to COVID-19 such as CTT have, in many ways, served to disrupt the false binary between the health system and community as separate entities interfaced solely through formal, often state-sanctioned structures such as health committees or community outreach programmes. Instead, what has emerged is the power of localised collective action in addressing many of the immediate health and socioeconomic challenges of the pandemic – often circumventing the “legitimate” avenues for action and engagement and resorting to informal networks and relationships instead.

## The Birth of Cape Town Together

 In early March 2020 a small group of public health folk, activists and community organisers (including the authors), saw the need for a collective, community-led response to COVID-19 in the city. As a result, CTT was born. The group began by putting together an online toolkit encouraging people to organise into a network of autonomous, self-organised, neighbourhood-level CANs. The underlying premise was that many of the challenges arising from COVID-19 – both epidemiological and social – are best responded to at the neighbourhood level. Usually beginning with a WhatsApp group, neighbours connect and assess the immediate needs of their specific community, identifying those who are more vulnerable and those who can volunteer to help.

Within two months over 170 CANs had emerged across the city. The CANs exist across the socioeconomic range of Cape Town’s extremely unequal neighbourhoods – from Khayelitsha, which has a population density of up to 16 500 people per square kilometre in some parts – to Constantia, one of the wealthiest suburbs in Cape Town, where the population density is 607 people per square kilometre.^3^ There is no copy-paste formula for the CANs, as each one develops according to the specificities of the neighbourhood. In some cases, pre-existing neighbourhood-level structures such as street committees or faith-based groups work alongside or together with the CAN. In other cases, completely new relationships were established both within and between neighbourhoods, often enabled through collaborations between CANs that have begun to connect across the social divides and legacies of segregation that still exist in the city.

 The CANs have received no financial support from the government, although most CANs engage in locally organised fundraising initiatives, raising money through donations within CANs or between CANs to generate resources. Under the CTT umbrella, there are multiple opportunities for the CANs to converge around various “nodes” in the network, offering spaces to share resources, knowledge, and to reflect and debrief on their experiences. There are also a number of thematic CANs working on resolving cross-network issues (such as building sustainable food systems, supporting neighbourhood-based alternatives for self-isolation, cross-network learning, and tackling fear and stigma of the virus), and a series of logistical teams doing fact-checking of health information, and designing informational materials for the network. The structure is de-centralised, non-hierarchical and self-organised and all neighbourhood CANs, thematic CANs and coordination teams are completely autonomous. New thematic CANs emerge organically on a regular basis in response to emerging needs, and old ones disintegrate as the energy of the group is needed elsewhere. There have been many challenges along the way, and not all the CANs operate seamlessly. This approach to organising is often met with confusion or suspicion, as it requires a leap of the imagination away from the hierarchical and highly structured forms of organising that many are used to. Nevertheless, at the time of writing in August 2020, the network had sustained for nearly 5 months with the occasional new CAN still emerging.

## Building Trust Through Building Relationships

 As the network has grown, the group has articulated ways of working to guide its development and facilitate its catalytic spread. These include foregrounding interpersonal connection and trust; an informal, adaptable structure, and collaboration with existing structures (including government) while remaining critical and resisting co-option. Building interpersonal relationships facilitates a large degree of trust, allowing for a dynamism that is unhindered by the need for constant accounting. This has also facilitated an ability to “move at the speed of trust.” This means using the strength of human relationships and mutual good faith to move quickly and without onerous accountability mechanisms whenever possible. When trust is absent, or when vested interests emerge, working at the speed of trust means slowing down to consider complexities, risks and fears, before taking any action.

 An example of this is the varied and highly effective network of community kitchens and food distribution schemes that are operated by the CANs. Food security quickly emerged as one of the most urgent issues within the CANs, as thousands of people already living on the edge of poverty lost their income as a consequence of South Africa’s decision to implement a hard lockdown. For a significant number of those facing massive food insecurity, the threat of hunger looms far greater than the threat of the virus itself. In response, most CANs now run multiple community kitchens and household food parcel distribution efforts, choosing how to do so based on local contextual factors that they are best-placed to assess and respond to. The immediate and widespread response of the CANs and the CTT network sharply contrasts with the slow, and lumbering response of the government to deliver state-sponsored food relief. In fact, well after lockdown began, food distribution through the CAN network and other civil society efforts remained the main source of food relief to a significant majority of people living in Cape Town. There has not been a systematic count of how many people the CANs are feeding, but an estimate established through self-reported data suggests that the number is well into the thousands.

 The experience of the CANs hints at the potential of a reimagined community health system in which the false binary between community and state is challenged, and the power of informal networks and collective action in unprecedented crisis situations is brought to the fore. But it has also brought into sharp focus the emergent tensions and complexities at this interface, even in the context of genuine commitment to engage from both communities and the formal health system.

A key tension relates to the core framings of the response to COVID-19. The government’s efforts to contain and mitigate the damage caused by the virus still focus predominantly on what science can tell us about how the virus spreads. As a result, resources are funnelled into a prioritised menu of public healthcare interventions that are formulated at higher levels of government by scientific command councils, based on particular framings of risk, and operationalised through standard operating procedures and bureaucratic implementation logics.^4^ Community intelligence – in other words, the tacit, situated knowledge arising from and produced within life-worlds and lived realities – cannot be compartmentalised into a standard operating procedure.^5^ Although fundamental to understanding the social spread of the virus, including the ways in which fear, stigma, poverty and the effects of lockdown intertwine to produce effects far more lethal than the virus itself – it has not been meaningfully incorporated into the state’s framing of the virus and its required response. A consequence of this is that, while this kind of knowledge is constantly generated and used by those at the community level, there is huge complexity in negotiating whether this knowledge could or should inform more of the state’s response.

As has been the experience of CTT, there is often a clash of very distinct ways of working when top-down and bottom-up approaches interface. To some degree, this can be overcome by building relationships and trust between actors, and there have been some remarkable examples of this kind of cooperation taking place, enabling the CANs to continue with their work with the tacit support of the state. The provincial Department of Health has recognised the network, attending some of the “co-learning” sessions that are hosted regularly. Additionally, there are a number of important “boundary spanning relationships” that have facilitated important links between the CANs and government. For example, there are many government employees who are also members of their neighbourhood CANs, as well as senior officials who are not CAN members but who understand their importance, allowing for a level of interconnection that serves to disrupt many of the usual bureaucracies that prevent these connections from forming organically.^6^

The challenge remains that the entrenched structure of a governmental department and an innately hierarchical approach to engaging with communities sometimes undermines the very principles that have made the CANs a central player in the COVID-19 response. This clash of approaches arises out of the fact that the CANs do not rely on hierarchies and bureaucracies to get the work done, but on building human connections. An imposition of a bureaucratised and hierarchical way of working – for example requiring a representative from the network who is “delegated to” from above, clashes with the principles of self-organising and decentralisation that enable the CANs to build relationships, take care of their neighbours, and operate autonomously and nimbly in their local context. While collaboration may well be important, its limits must be recognised – particularly in the context of an unprecedented crisis where all systems are being pushed to capacity. It is important that bottom-up movements practicing a decentralised, self-organised approach, are able to resist the tendency to default to command and control approaches – especially when pressure increases and there is a desire to “scale up.”^7^ It is also important to resist the threat of depoliticization, where participatory projects become “tools of governance that contribute to the maintenance of the status quo, rather than serving as strategies for resistance and change by excluded groups.”^8^

The experience of CTT as an emergent community-based response to the pandemic in Cape Town – a city plagued by chasmic economic inequalities and social division – is an example of the necessary growth of bottom-up mutual aid initiatives in the stark absence of a state-led social safety net. It demonstrates the power of informal networks and invented spaces, which are able, with limited material and financial resources, to provide widespread social support at a neighbourhood level in times of crisis.^9,10^ In moving towards a more coherent and trust-based interface between such initiatives and the formal health system it is imperative that boundary spanning operates in a bi-directional manner. Engagement must be sustained over time, rather than once-off meetings and check-box style public participation. There is also a need for recognition, on the part of the state, of the work of informal networks and a willingness to resource and support this work without making it dependent on formalisation and bureaucratisation.

 CTT has demonstrated that many of the people who play a vital role in the community health system are not necessarily recognised as being part of it – the women who organise scarce resources to cook for their neighbours in need, the person with phone numbers for everyone on their street, the retired nurse and grandmother who is called upon for medical advice, or the health official who is also a community member when she returns home from work – these have been some of the most prominent players of Cape Town’s community health response to COVID-19. As the rise of mutual aid initiatives across the world shows, the seeds of collective action and self-organising have always existed. The experiences of community mobilisation in the face of a threat such as COVID-19 provide important opportunities for re-thinking community health systems, in particular the challenges of sustaining collective action that is community-initiated and -driven, rather than state organised. A foundation has been laid to build back better, towards a world in which community-led responses have an important and legitimate role to play.

## Acknowledgements

 Helen Schneider, School of Public Health, University of the Western Cape, Cape Town, South Africa, who contributed towards revision of the manuscript for important intellectual content and overall supervision.

## Ethical issues

 Not applicable.

## Competing interests

 MVR reports that the authors are active, volunteer participants in the CTT movement.

## Authors’ contributions

 All authors contributed to the conceptualisation and writing of the piece.

## Authors’ affiliations

^1^School of Public Health, University of the Western Cape, Cape Town, South Africa. ^2^Health Policy and Systems Division, School of Public Health and Family Medicine, University of Cape Town, Cape Town, South Africa.
